# *Plasmo*GEM, a database supporting a community resource for large-scale experimental genetics in malaria parasites

**DOI:** 10.1093/nar/gku1143

**Published:** 2014-12-12

**Authors:** Frank Schwach, Ellen Bushell, Ana Rita Gomes, Burcu Anar, Gareth Girling, Colin Herd, Julian C. Rayner, Oliver Billker

**Affiliations:** Wellcome Trust Sanger Institute, Hinxton Cambridge, CB10 1SA, UK

## Abstract

The *Plasmodium* Genetic Modification (*Plasmo*GEM) database (http://plasmogem.sanger.ac.uk) provides access to a resource of modular, versatile and adaptable vectors for genome modification of *Plasmodium* spp. parasites. *Plasmo*GEM currently consists of >2000 plasmids designed to modify the genome of *Plasmodium berghei*, a malaria parasite of rodents, which can be requested by non-profit research organisations free of charge. *Plasmo*GEM vectors are designed with long homology arms for efficient genome integration and carry gene specific barcodes to identify individual mutants. They can be used for a wide array of applications, including protein localisation, gene interaction studies and high-throughput genetic screens. The vector production pipeline is supported by a custom software suite that automates both the vector design process and quality control by full-length sequencing of the finished vectors. The *Plasmo*GEM web interface allows users to search a database of finished knock-out and gene tagging vectors, view details of their designs, download vector sequence in different formats and view available quality control data as well as suggested genotyping strategies. We also make gDNA library clones and intermediate vectors available for researchers to produce vectors for themselves.

## INTRODUCTION

Malaria is caused by parasites of the genus *Plasmodium*, members of which have adapted to infect a wide range of vertebrate hosts. It remains one of the most important infectious diseases of humans, and the repeated evolution of parasites that are resistant to front-line antimalarial drugs makes the identification of new drug and vaccine targets an urgent priority. In the past two decades, experimental genetic approaches have been employed by numerous labs to analyse gene function and assess target suitability. However, while progress has been made, obstacles to systematic genetic approaches remain. Foreign DNA can be introduced by electroporation while the parasites are haploid and replicate asexually in erythrocytes. Recombination in *Plasmodium* is almost exclusively homologous, but even in the most tractable species, the rodent parasite *P. berghei*, recombination rates using conventional technologies are low (usually not exceeding 10^−3^) (1). The extremely high content of adenine and thymine nucleotides (>77%) poses significant additional challenges to scaling up manipulation of the genome because it renders *P. berghei* genomic DNA unstable in *E. coli*. The latter problem can be overcome in part by using a new class of low copy linear plasmid derived from phage N15 (2), which has allowed the generation of a representative genomic library covering most *P. berghei* genes in their entirety to be maintained in *E. coli* for the first time (3). We have recently established protocols that use *lambda* phage recombinase-mediated engineering (4,5) to convert genomic DNA clones from an arrayed library into genetic modification vectors. Due to their longer homology arms, these vectors integrate into the genome with enhanced efficiency (3,6).

Since recombineering technology is robust and scalable, but not generally established in parasitology laboratories, we have initiated the *Plasmodium* Genetic Modification Project (*Plasmo*GEM), which has at its heart a production pipeline for a genome wide community resource of barcoded genetic modification vectors. Most users currently request *Plasmo*GEM vectors to speed up conventional small- to medium-scale gene targeting or tagging projects, but vectors can also serve as starting points for more complex conditional alleles or allelic exchange vectors (7–9). An important advantage of building a global resource is that each vector is provided with a gene-specific molecular barcode which may be used to identify individual mutants.

To enable the production of thousands of genetic modification vectors, we have generated a suite of software tools that automate vector design, assign gene specific molecular barcodes, guide the vector production process in the laboratory, carry out quality control on each vector using next-generation sequencing and finally suggest strategies for genotyping genetically modified parasites. Here, we describe the software and database that support the *Plasmo*GEM production pipeline and that provide public access to the resource.

## DESCRIPTION OF THE RESOURCE AND DATABASE

### *Plasmo*GEM vectors provide a modular toolkit for genome manipulation

**Figure 1.**
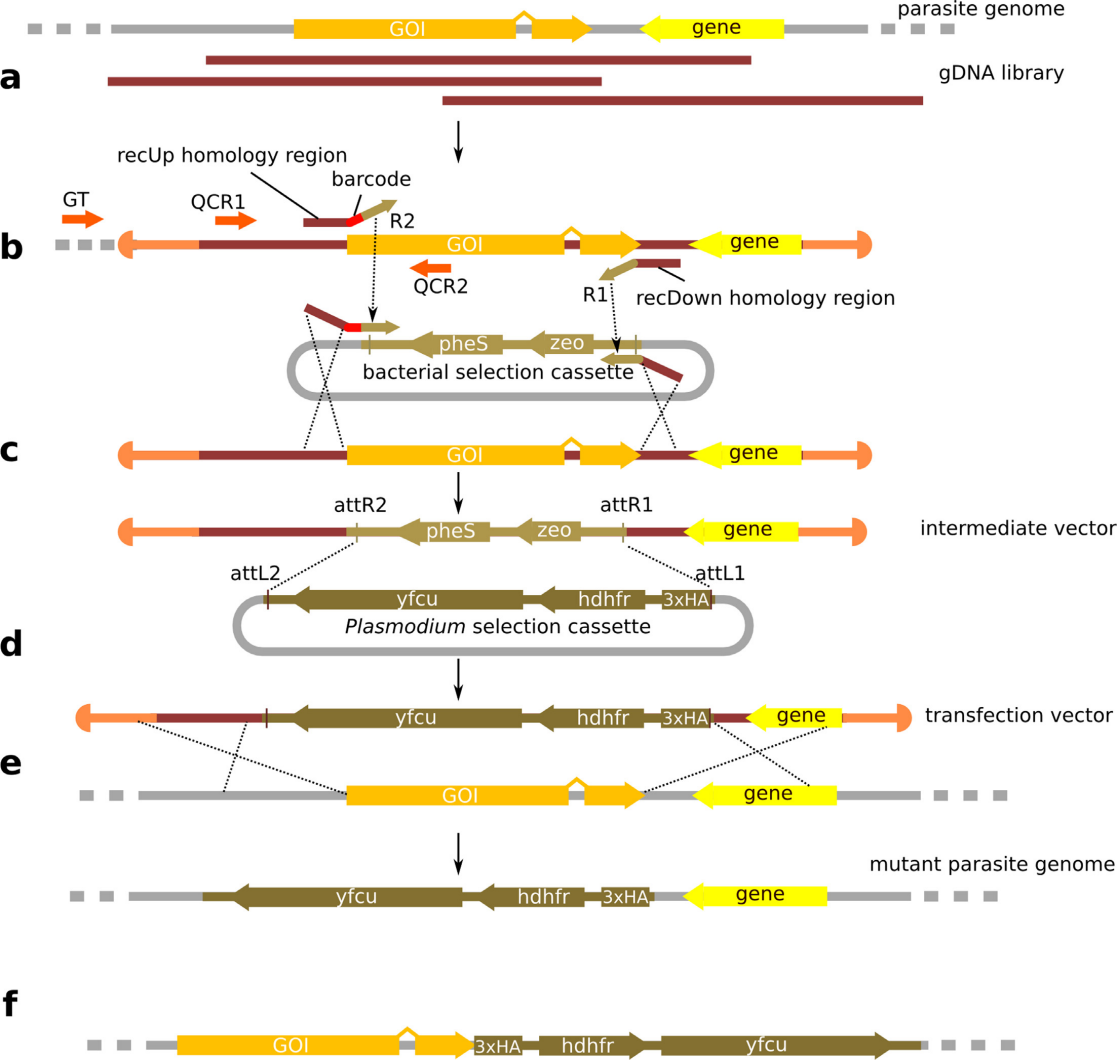
Graphical overview of vector design. To produce each vector, a suitable library clone, if possible with full coverage of the gene of interest (GOI), is picked by the software (**a**). After adding sequence annotations to the library clone, two 50 nt homology regions are selected towards the 5' and 3' end of the GOI (recUp/recDown) for recombinase-mediated engineering. These are assembled into recombineering oligomers by adding a barcode module to recUp and the R1/R2 PCR primer 3' annealing sequences (**b**). A pair of quality control PCR primers (QCR1/QCR2) and a genotyping primer (GT) are also designed. The recUp/Down pair is used to amplify the bacterial selection marker cassette, thus adding the 50 nt *Plasmodium* genomic sequences to the cassette to drive the Red/ET recombinase mediated replacement of the GOI, which gives rise to the intermediate vector (**c**). A Gateway reaction swaps the bacterial for a *Plasmodium* selection marker cassette to produce the final transfection vector (**d**). The final vector is transfected into *P. berghei* schizonts and pyrimethamine selects for genomic integration by homologous recombination (**e**). For C-terminal peptide tagging designs the process is similar, except that only the stop codon of the GOI is replaced by the selection marker, which fuses a marker peptide (here a triple HA tag) to the GOI in frame (**f**). From the unmodified state to the final transfection vector, library clone inserts are maintained in the linear pJazz vector backbone.

The growing *Plasmo*GEM resource currently consists of 1857 vectors for the deletion or disruption of *P. berghei* genes (covering ∼1/3 of all protein coding genes), 255 vectors for C-terminal tagging with a triple haemagglutinin (3xHA) tag, and 9113 gDNA library clones that provide partial or complete coverage of 95% of all *P. berghei* ANKA genes. Typical vector designs are illustrated in Figure 1. The starting material for all *Plasmo*GEM vectors is a gDNA library clone mapping to the target gene or its regulatory elements. Vector production proceeds in two stages (3). For knock-out vectors a region of the library clone is first replaced by a bacterial selection cassette using *lambda* Red-ET recombinase-mediated engineering (4,5), then the resulting intermediate plasmid is turned into a transfection vector by a Gateway recombinase reaction that exchanges the bacterial selection cassette with the final markers for selection in *P. berghei*. For tagging vectors, the bacterial selection marker is inserted immediately upstream of the stop codon, such that after the Gateway reaction the 3xHA tag is fused in frame to the gene of interest, followed by a generic terminator sequence from the *P. berghei dhfr-ts* gene (Figure 1f). While these two vector designs are now in production, the approach is adaptable, and Gateway entry clones can be built to enable the Gateway reaction to have a range of outcomes, from inserting alternate tags to creating more complex mutations such as promoter swap or allele exchange. A growing panel of Gateway entry clones for diverse protein tags are also available through the resource, and users can request intermediate vectors, of which 2107 can currently be supplied, to customise gene modification vectors according to their specific needs.

### The *Plasmo*GEM database

**Figure 2.**
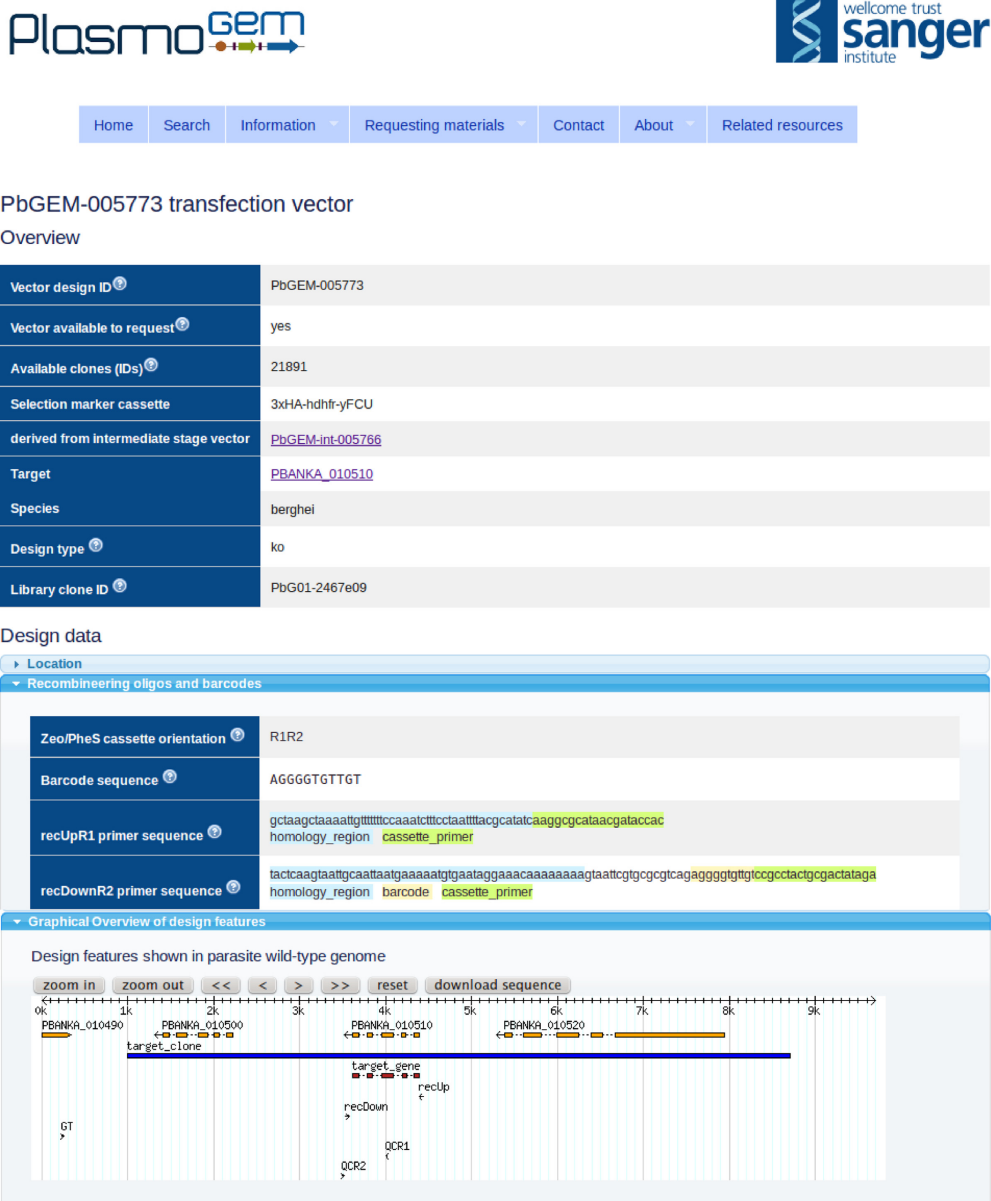
Screenshot showing a design data page of a knock-out vector for PBANKA_010510, a conserved *Plasmodium* protein of unknown function. Sequences of the recombineering and quality control oligomers are given and interactive genome-browser-like widgets present graphical representations of the design and the final mutated locus in the parasite genome. Fully annotated sequences of the vector designs, the intermediates and final transfection vectors can be downloaded in Genbank format from here.

Public access to all *Plasmo*GEM resources is available at http://plasmogem.sanger.ac.uk through a web application written in the Perl Catalyst framework. To find available resources users can browse the genome or search by keywords, gene name or gene identifier. Searches can include the whole genome, or be restricted to genes for which resources are already available. Vector designs are generated using an automated approach (see below), and for each design users can drill down to a detailed data page with graphical representations and links to downloadable Genbank files for both the targeting vector and the modified target locus that will be generated upon insertion of that vector (Figure 2). Sequences of oligonucleotide primers suitable for validating the identity of the vector and for genotyping the genetically modified parasite are suggested. Different designs may have different utilities for genetic modification, and users are made aware of risks associated with particular designs. Users can drill down to quality control data from full-length deep sequencing of homology arms for up to four clones implementing any given vector design. In cases where no sequence-perfect clone was obtained, the quality control pages display graphical representations showing types and locations of any mutations to enable users to make informed choices of which clone to select.

### Automated vector design

The MySQL database that is accessed through the web interface also underpins an information management system that controls the vector production pipeline. The database is populated by a custom software suite, written in Perl, which automates vector design, sample management and quality control. The design process begins with the selection of the most suitable gDNA library clone. This is followed by annotation of all relevant genomic features including genes and putative regulatory elements, based on the *P. berghei* ANKA reference genome available from the GeneDB FTP page ftp://ftp.sanger.ac.uk/pub/pathogens/P_berghei/January_2011. Target sites, 50 nt in length, are then selected for recombinase-mediated engineering of the intermediate vectors and used to design two synthetic oligonucleotides for the recombineering step, one of which includes a gene specific barcode module.

Clone selection is designed to increase recombination frequency by favouring library clones with long homology arms either side of the target gene. However, we noticed a sharp drop in the overall success of the production pipeline with library clone inserts above 10 kb (Supplementary Figure S1). For that reason, library clones less than 10 kb in length are preferred by the selection algorithm. *Plasmodium* genes have relatively few and mostly small introns, meaning that knock-out vectors can often be designed to delete the entire protein coding sequence of the target gene. However, in many vectors the deleted region is actually smaller than the complete gene, for instance when no gDNA clone provides complete coverage or to ensure homology arms are at least 1 kb long. The *Plasmodium* genome is relatively compact, meaning that regulatory and UTR regions can overlap between neighbouring genes. Deletion vectors are therefore designed not to interfere with 1 kb upstream and 0.8 kb downstream of neighbouring open reading frames. Data from strand-specific RNA sequencing may soon reveal the structure of individual transcripts more precisely and may allow these rules to be refined for future vector designs. The current decision tree that underpins clone selection and vector design is illustrated in Supplementary Figure S2.

### Molecular barcodes

Each *Plasmo*GEM vector carries a molecular barcode consisting of 11 variable nucleotides flanked by constant primer annealing sites for PCR amplification. Barcodes can be used to identify individual vectors and transgenic parasites, but their main application is for quantitative analyses using barcode counting (10) on a benchtop sequencer. Analysing mixed populations of mutants in this way will enable drop out screens and will make it possible for the first time to measure individual growth rates within parasite pools composed of different mutants in the same mouse.

The barcode module is included in one of the synthetic oligonucleotides used for the recombineering step of vector production. Most barcodes are gene rather than vector-specific, i.e. tagging and knock-out vectors for the same gene usually share the same barcode. They are assigned from a list of 5300 unique 11mers with a Hamming distance of at least four, i.e. sequences are unique with up to four base miscalls between any two barcodes. This level of uniqueness prevents cross contamination of barcode counts because single nucleotide changes or sequencing errors can be detected and corrected while double errors can be detected and discarded at the analysis stage. A few vectors with Hamming distances <4 were initially created and are flagged up on the vector pages of the database. A constant pair of PCR primers can be used to amplify the barcode module from all *Plasmo*GEM constructs.

### Quality control by next-generation sequencing

**Figure 3.**
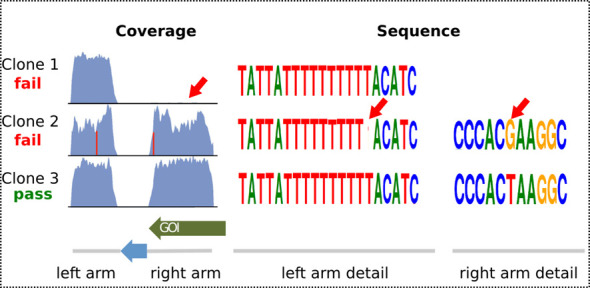
Quality control by next-generation sequencing of vector PbGEM-040545 (C-terminal tagging of PBANKA_110360, ATPase3), illustrating use of coverage to detect loss of the right homology arm in clone 1, and of sequence alignments to detect a single nucleotide deletion in the left arm and a point mutation in the right arm of clone 2.

The highly AT rich *P. berghei* genome contains many repetitive sequences and long homopolymeric tracts of adenine and thymine nucleotides that accumulate mutations at an increased rate when propagated in *E. coli*. Some of these could inadvertently modify neighbouring genes, if introduced into the parasite genome by the long homology arms of recombineered vectors. In the worst case (and in the absence of genetic complementation experiments) this may lead to the misattribution of a phenotype to the wrong gene. The homology arms and barcodes of vectors are therefore verified at the end of the production pipeline by sequencing four clones per design on an Illumina MiSeq instrument (150 bp paired ends are sequenced of 400–600 bp fragments (11)). This approach proved economical since production plates are laid out to eliminate overlap between homology arms of different vectors, allowing reads from a sequencing library encompassing an entire plate to be mapped back unambiguously to individual vectors. We typically sequence four colonies each from five plates together using different Illumina index codes. Read mapping and alignment processing uses open-source tools SMALT (https://www.sanger.ac.uk/resources/software/smalt/) and SAMtools (12). Mutations are called by a module of our custom software suite. Only high-quality alignments (quality score ≥ 20) within the expected insert-size range are used for the analysis. Point mutations and short insertions or deletions, which most often occur in long homopolymeric tracts of A or T nucleotides, are called when Binary Alignment/Map (BAM) files (12) reveal at least 25% of aligned reads with the mutation. Larger mutations, such as loss of a homology arm or structural rearrangements, are detected from drops of read coverage below 30% of the average depth (Figure 3).

Vectors fail quality control if they carry mutations in open reading frames of the target gene (tagging vectors) or any neighbouring gene (all vectors), regardless of whether the mutation would be synonymous or not. Mutations in the barcode module or longer deletions and rearrangements also fail quality control. Where no sequence-perfect clone is available, point mutations or small insertions/deletions in non-coding regions are tolerated and flagged in the database so that users are aware of them and can make informed choices about vector usage. Where we suspect natural deviations from the reference genome, such as occur at naturally polymorphic loci, automated quality control can be overridden by a curator.

### Mutant genotyping

The vector design software integrates the Primer3 suite (13) to generate sequences for three gene-specific PCR primers for genotyping. Parameters used for primer design are shown in Supplementary Table S1. Primer pair QCR1/QCR2 (Figure 1) produces a 300–700 bp product across the intended insertion/deletion site. They are used initially to verify the identity of the library clone at the start of vector production. During genotyping of mutant parasites they serve to detect the unmodified genomic locus. Combined with constant primers annealing in the intermediate and final cassettes, QCR2 is used again to monitor the production process and to genotype transgenic parasite clones. A third primer, designated GT (for genotyping), anneals to the genomic sequence outside of the region covered by the gDNA clone. It is used in combination with one of the constant primers binding to the *P. berghei* selection cassette to verify the integration of the construct into the parasite genome. This PCR product is designed to span the shorter of the two homology arms.

### Integration with other Databases

The availability of *Plasmo*GEM library clones andgene modification vectors is exported to PlasmoDB (http://plasmodb.org), the *Plasmodium* community database, on a daily basis and displayed on PlasmoDB gene data pages and search results. *Plasmo*GEM gene pages link to corresponding entries on GeneDB (http://www.genedb.org) and the Rodent Malaria genetically modified Parasites database (RMgmDB; http://www.pberghei.eu). Vice versa, RMgmDB now links back to *Plasmo*GEM for genetic modifications introduced by *Plasmo*GEM vectors. We encourage all recipients of our vectors to submit phenotypes to RMgmDB.

### Availability

All data can be freely accessed via the *Plasmo*GEM website at http://plasmogem.sanger.ac.uk. We welcome user feedback, including requests for new vectors to be produced, by email to plasmogem@sanger.ac.uk. Materials can be requested by non-profit organisations free of charge. These include finished gene modification vectors and unmodified library clones as well as vectors required to turn wild-type clones into gene modification vectors following our published protocols (14). Details for the request procedure are available at http://plasmogem.sanger.ac.uk/request/howto.

## DISCUSSION AND FUTURE PLANS

The *Plasmo*GEM database makes a growing resource of genetic modification vectors and design tools accessible to the research community. These can greatly reduce the time and effort needed to delete or modify parasite genes, thereby allowing researchers to apply conventional approaches to larger numbers of genes more effectively. Importantly, gene specific sequence barcodes will open up new approaches that rely on barcode counting, such as competitive growth phenotyping of multiple mutants in the same mouse. Recording phenotype data for individual vectors in the database will be a major future addition.

Currently, the *Plasmo*GEM resource already covers about one third of all *P. berghei* genes with knock-out constructs. We will grow the resource further to include the vast majority of protein coding genes for which suitable genomic library clones are available, perhaps with the exception of large families of variant surface antigens.

The *Plasmo*GEM database will be developed to accommodate a growing number of functionally diverse selection cassettes for gene deletion and 3' tagging, but we are currently focussing on gene deletion vectors for *P. berghei*, in part because nearly 50% of the genes in the *Plasmodium* genomes have no annotated function or homologue outside closely related parasite species. Importantly, the *Plasmo*GEM software and production pipeline can readily be pointed at other *Plasmodium* genomes to perform similar tasks.

A key question will be how to focus future efforts, and while full genome coverage is desirable, genome-wide sets of vectors will not be possible for all construct designs and species. Community involvement in the direction and uptake of the resource will therefore be key, and we plan to adapt the website to include an online ordering system and the ability to request genes to be targeted by new *Plasmo*GEM vectors. In the meantime, we encourage our users to send us suggestions for new vectors via email to plasmogem@sanger.ac.uk. Community annotation of vector pages, recording which ones have been used successfully in what circumstances, would also be a useful feature, with basic phenotyping data linked through to the RMgmDB.

Community-led large-scale experimental genetic projects have revolutionised the understanding of model organisms from yeast to *Drosophila* to mice. We hope that *Plasmo*GEM will serve a catalysing function for a similar community revolution in *Plasmodium* experimental genetics.

## SUPPLEMENTARY DATA

Supplementary Data are available at NAR Online.
